# The impact of an organizational-level mindfulness-based intervention on workplace social capital and psychological safety: A qualitative content analysis

**DOI:** 10.3389/fpsyg.2023.1112907

**Published:** 2023-03-07

**Authors:** Emilie Hasager Bonde, Eva Gemzøe Mikkelsen, Lone Overby Fjorback, Lise Juul

**Affiliations:** ^1^Danish Center for Mindfulness, Department of Clinical Medicine, Aarhus University, Aarhus, Denmark; ^2^Department of Psychology, University of Southern Denmark, Odense, Denmark

**Keywords:** workplace, psychosocial work environment, mindfulness, mental health promotion and prevention, qualitative methods, social capital, psychological safety

## Abstract

**Background:**

Through the past decades, the mental health of the European population has been continuously declining. Social relations in various spheres of life, including workplace settings, have been shown to impact mental health. Mindfulness-based stress reduction (MBSR) has been found effective in enhancing well-being, and reducing perceived stress, and symptoms of depression and anxiety. Research into mindfulness-based interventions (MBIs) in workplace settings has shown that these interventions may positively affect workplace outcomes, such as interpersonal relations. However, research regarding the organizational impacts of MBIs is still nascent. The objective of this study was to investigate how an organizational-level mindfulness-based intervention (MBI) including a workplace-adapted MBSR programme may impact workplace social capital and psychological safety.

**Methods:**

Four small and medium-sized private companies were included in this study, representing 368 employees and managers. The intervention consisted of three steps: 1. Mandatory participation in introductory sessions on mental health and mindfulness, 2. Voluntary participation in a 10-week workplace-adapted MBSR programme, and 3. A workshop for selected employee representatives and managers on further implementation of mindfulness. Data was collected using pre and post-intervention focus group interviews. In total, 27 interviews including 76 respondents were conducted. Verbatim transcription was performed. Data was analyzed using deductive content analysis with theoretical frameworks for social capital and psychological safety.

**Results:**

The analysis resulted in three main categories: 1. Social capital (1.1. bonding social capital, 1.2. bridging social capital, 1.3. linking social capital), 2. Psychological safety, and 3. Emergent theme: The role of lockdown on the perceived organizational impact of a workplace MBI. The greatest impact was found relating to the bridging social capital, i.e., social capital between departments, and psychological safety among colleagues at the same level of employment.

**Conclusion:**

The results indicate that company participation in this organizational-level MBI including a workplace-adapted MBSR programme may positively impact social relations at work, especially the bridging social capital and psychological safety between colleagues at the same level of employment. These results may have been influenced by lockdowns due to the COVID-19 pandemic.

## 1. Introduction

The mental health of the European population has been eroding through the past decades (WHO, 2018), and data from the Global Burden of Disease demonstrate a global increase in disability adjusted life years (DALYs) due to mental disorders during the past 30 years (GBD Mental Disorders Collaborators, 2022). Previous research has shown social relations to be of great importance to mental well-being (Roffey, 2021). As such, positive social relations are associated with higher levels of well-being, and have been seen to have a buffering effect on mental disorders, such as anxiety (Teo et al., 2013) and depression (Santini et al., 2015). Conversely, negative social relations are associated with poor mental health outcomes and ultimately higher mortality (Holt-Lunstad et al., 2010). People engage in social relations of shorter or longer duration in a multitude of settings, including the workplace. Of the World’s population, about 60% are part of the work force (ILO, 2022). Thus, interpersonal relationships in workplace settings are likely to affect the well-being of a large part of the World’s population. Indeed, previous research has shown that negative social elations at work, such as interpersonal conflict, workplace bullying, social isolation, and a lack of social support pose a serious threat to the mental health of employees and managers (Mikkelsen et al., 2020; WHO, 2022). Accordingly, there is a potential preventative and health promoting gain by implementing interventions that may ameliorate or enhance social relations in workplace settings.

A relevant research area to look to when aiming to positively affect social relations in workplace setting is workplace social capital. The concept of social capital refers to “[…] *features of social organisation such as networks, norms, and social trust that facilitate coordination and cooperation for mutual benefit*” (Putnam, 1995). Research shows that low workplace social capital may be associated with decreased well-being, and increased psychological stress and depression (Pattussi et al., 2016). Thus, impacting workplace social capital may potentially affect employees’ and managers’ mental health positively.

Woolcock and Narayan (2000) and Szreter and Woolcock (2004) divide social capital into three categories; bonding social capital, bridging social capital, and linking social capital. Bonding social capital refers to the social capital within a group with a shared social identity, for example, a team. Bridging social capital refers to the horizontal social capital between groups, for example, two different departments within the same organization. Linking social capital refers to the vertical social capital between people who engage in interactions characterized by a formal or informal difference in power, for example, managers and employees (Szreter and Woolcock, 2004).

The concept of social capital is multi-facetted entailing both networks, norms, and trust as key-features affecting social organizations such as workplaces. *Networks* refer to “*ties that people and organizations use over time to get access to the resources they need*” (Schneider, 2009), *norms* are the normative way of doing things in an organization (Olesen et al., 2008), and trust is the willingness to be vulnerable, based on the expectations that others will react favorable to this vulnerability (Edmondson, 1999). According to Edmondson (2004), as a descriptor of the quality of interpersonal relations, trust relates to longer time perspectives, for example, several weeks from a given time point. Thus, trust entails a general feeling of trust in others to behave in a certain way, for example, to behave favorably to individual displays of vulnerability (Edmondson, 2004). However, the level of trust in a given workplace relationship does not necessarily offer insights into employees’ or managers’ feelings of being psychological “safe” in specific situations, for example, feeling safe that one will not be scolded for blunders at work or for raising a difficult issue (Edmondson, 2004).

When studying shared feelings of being psychological safe within given work-spheres, the concept of *psychological safety* may be employed. Psychological safety concerns the individual or shared feeling of how others (e.g., co-workers) will react when difficult subjects are raised, blunders are made, or someone suggests a different way to approach a work problem (Edmondson, 2004). As such, psychological safety is defined as “*individuals’ perceptions about the consequences of interpersonal risks in their work environment*” (Edmondson, 2004). Hence, in groups with a high degree of psychological safety, group members, such as employees and managers within a department, would for example, feel safe to suggest new ways of doing things, and admitting to blunders. On the contrary, in groups with a low degree of psychological safety, group members may fear admitting to blunders or providing feedback to other team members. Thus, the concept of psychological safety may contribute to a more in-depth understanding of the general level of trust within an organization that might be captured by solely investigating workplace social capital.

Improving the workplace social capital and psychological safety and thereby enhancing mental well-being may be approached in a multitude of ways. Mindfulness-based interventions (MBI) have been found to be among the most effective psychological interventions to improve mental well-being (van Agteren et al., 2021). Mindfulness is defined as *“… the awareness arising through paying attention on purpose in the present moment, non-judgmentally, in the service of self-understanding, wisdom, and compassion*” (Kabat-Zinn, 2018). Previous research has also demonstrated MBIs in workplace settings to be effective in enhancing well-being as well as reducing perceived stress and self-reported symptoms of depression and anxiety (Vonderlin et al., 2020). Furthermore, a recent integrated review of the effect of mindfulness in the workplace demonstrated effects on both work-related well-being and organizational outcomes, such as enhanced leadership qualities and better interpersonal relationships (Panditharathne and Chen, 2021). On the basis of findings from previous research, the World Health Organization (WHO) mentions 5 MBIs as potential beneficial interventions to strengthen mental health in the workplace in their recent publication from Autumn 2022 (WHO, 2022).

Where the effects of MBIs on the mental health of individuals are well-documented (De Vibe et al., 2017; Panditharathne and Chen, 2021; van Agteren et al., 2021), less in known about the potential impact of such MBIs on entire organizations. However, based on the evidence of effects of MBIs on individuals, Good et al. (2015) propose mindfulness to be effective in improving the psychosocial work environment. Being purposefully aware in the present moment allows individuals to notice when the attention is wandering and to kindly bring the attention back to the present moment (Dahl et al., 2020). In a study by Killingsworth and Gilbert (2010), the authors find that humans are only mentally present in what they are doing approximately half of the time (Killingsworth and Gilbert, 2010). Being on this mental time travel may have consequences for individuals’ relationships. When one is unaware of thoughts, feelings, or mood, this may lead to automatic reactions and to not responding constructively to any given social situation (Kabat-Zinn, 2013; Crane et al., 2017). For example, one could be thinking about other things, while having a conversation, which would prevent one from really listening to the other person. Practicing mindfulness may cause a shift in how individuals relate to, e.g., their thoughts, perceptions and feelings as well as to outer circumstances, including social relations (Crane et al., 2017). This competence is called meta-awareness (Dahl et al., 2020). Meta-awareness may enhance individuals’ possibility of responding more skillfully. Thus, when an individual is aware of his or her physical and emotional state, it allows for a greater awareness and understanding of others and how others act (Glomb et al., 2011). In social situations, these competencies may enable individuals to listen more actively and not get distracted, for example, during a conversation (Dahl et al., 2020), or to respond in a reflected manner instead of automatically reacting (Kabat-Zinn, 2013). Accordingly, in an integrative review on mindfulness and social sustainability, Sajjad and Shahbaz (2020) found that mindful individuals may affect workplaces at an organizational level by means of enhanced pro-social behavior and improved interpersonal relationships (Sajjad and Shahbaz, 2020). Proposed mediators of the association between mindfulness and interpersonal relations are, for example, reduced number conflicts, improved communication and higher levels of empathy and compassion (Good et al., 2015). Hence, mindful individuals may impact interpersonal relationships in the workplace (Sajjad and Shahbaz, 2020; Panditharathne and Chen, 2021). As workplace social capital and psychological safety are both social, interpersonal constructs, changes in relationship quality following an MBI may be reflected by changes in the workplace social capital and psychological safety.

Mindfulness-based stress reduction (MBSR) is an 8-week curriculum-based programme delivered by a trained MBSR teacher. The programme entails a total of nine sessions: eight weekly 2.5 h sessions and one 7-h silent retreat day. Importantly, MBSR is a group-based intervention delivered in groups of up to 30 individuals. The MBSR programme includes experience-based knowledge of, for example, how people perceive social situations differently, and the ability to view challenging interactions from the other person’s perspective (Mccown et al., 2010; Kabat-Zinn, 2013; Santorelli, 2014). Moreover, throughout the 8-week programme, the MBSR teacher focusses on creating a safe and trusting group environment. We propose that this explicit focus on a safe and trusting environment for sharing ones experiences may have an independent influence on the workplace social capital and psychological safety as trust and safety are key elements of these two theoretical concepts (Putnam, 1995; Edmondson, 1999). According to the Medical Research Council, to support a mental health promoting environment, interventions may effectively target entire organizations and not merely selected groups within organizations (Skivington et al., 2021). By implementing interventions at an organizational-level, these interventions may facilitate system change, and hence result in healthier work environments (Skivington et al., 2021). Based on theoretical assumptions and findings from previous research, the purpose of the present study was to investigate if and how the social capital and psychological safety may be impacted by company participation in an organizational-level MBI including a workplace-adapted MBSR programme. Specifically, we propose that organizational participation in an MBSR programme may affect the psychosocial work environment through improvements in interpersonal relationships in the workplace. We propose that these improvements may be brought about by enhanced awareness of, e.g., one’s own thoughts, feelings, and mood, patterns of reaction, and attention to others. Moreover, we propose that the explicit focus on creating safe environments in the MBSR programme may impact interpersonal relationships and thus influence the workplace social capital and psychological safety. Therefore, two research questions relating to how mindfulness may affect interpersonal relationships in the workplace were explored: (1) how might the organizational norms, networks, and trust be impacted by an organizational-level, mindfulness-based intervention?, and (2) how might this intervention affect employees’ and managers’ perception of safety regarding interpersonal risk-taking?

## 2. Methods

### 2.1. Design

The present qualitative study was part of a quasi-experimental multi-method trial that investigated the feasibility and impact of implementing workplace-adapted MBSR at organizational level in small or medium-sized Danish companies. The present study concerns the interpersonal impact. Prior to commencement, the trial was registered with the Danish Data Protection Agency (2016-051-000001/1715).

### 2.2. Participants and recruitment

To be eligible for inclusion in the trial, companies had to be small or medium-sized companies (SMEs), with 10-249 employees and managers, either partly or entirely based in Denmark. To enroll, top management in each company had to consent to the employees and managers participating in the intervention during working hours, or alternatively give monetary compensation for the time spend participating outside working hours.

In total, four SMEs enrolled in the trial, each representing a different business area: Media Company, chain of restaurants, Production Company, and an IT-company. Company 1–3 represent companies based entirely in Denmark, while Company 4 is based partly in Denmark but operates with offices worldwide.

Multi-channel recruitment of the companies were conducted using digital newsletters from trade organizations, direct contact to seemingly relevant SMEs, social media posts on LinkedIn, Twitter and Facebook, and posts on the Danish Center for Mindfulness’s webpage. Recruitment were ongoing from January 2020 to October 2020. When a company expressed interest in participating, an initial meeting was held between project manager, the last author LJ, an MBSR teacher and representatives from the company management. At this meeting, the company representatives were informed that the intervention was to be at an organization level. Hence, participation in the intervention had to be offered to all employees and managers, and not solely offered to selected groups. Furthermore, the company management were informed that, as an obligatory part of the intervention, all employees and managers were to participate in a two-hour information session during working hours. Moreover, LJ emphasized the requirement that all employees and managers should have the opportunity to participate in a 10-weeks live online MBSR programme during working hours or alternatively receive compensation for the time spend outside working hours. Upon acceptance of these terms, a contract was signed by a company representative, most often a representative of the top management.

### 2.3. Intervention

The intervention in this study was a workplace-adapted MBI in three steps: (1) an obligatory two-hour introductory session concerning mental health and mindfulness for all employees and managers in each company. (2) Participation in a 10-weeks workplace-adapted live online MBSR programme delivered *via* Zoom to all self-selected employees and managers. (3) A workshop on further implementation of mindfulness in the companies for selected employee representatives and managers.

The two-hour introductory sessions were held either live online *via* Zoom (Company 1, 3 and 4) or at a company site (Company 2) according to the company’s preference. The sessions consisted of a power point presentation regarding mental health, stress, mindfulness and research within this area. Furthermore, employees and managers were invited to engage in a brief seated meditation and standing yoga practices during the introductory sessions. At the end of the sessions, employees and managers were offered the opportunity to sign up for participation in a 10-weeks workplace-adapted MBSR programme. The purpose of the obligatory introductory sessions was to provide information about mental health and mindfulness and to ensure that all employees and managers received the same information about the intervention.

To secure that the workplace-adapted MBSR programme entailed all active components of the original MBSR programme, while also aiming for optimal contextual fit, adaptations were made using Crane et al.’s (2017) framework for adapting MBIs to new contexts and/or populations. Hence, the content of this workplace adapted MBSR programme was structured according to the MBSR curriculum. However, the duration of the programme was 10 weeks with weekly 1.5 h sessions. Adaptations from the original MBSR programme to the workplace-adapted MBSR programme is illustrated in Table 1. A trained MBSR teacher delivered the 10-weeks workplace-adapted MBSR programme live online *via* Zoom to groups of 5–22 managers and/or employees. In two out of four companies, employees and managers were divided into different groups. In the other two companies, this division was either not feasible due to a small number of managers, or because of a request made by the company to have mixed employee-manager groups. The MBSR teaching includes an experienced-based learning approach, where participants are invited to practice mindfulness through, for example, meditation, body scan and yoga practices. Moreover, the MBSR teachers engage participants in inquiry regarding direct experiences during these mindfulness practices (Crane et al., 2017). To ensure fidelity, the third author (LOF) supervised all MBSR teachers who delivered one or more of the 10-weeks workplace-adapted MBSR programmes throughout the intervention. Supervision was done according to the Mindfulness-based interventions: teaching assessment criteria (MBI-TAC; Crane et al., 2021).

**Table 1:** Structural differences between the original MBSR programme and the workplace-adapted MBSR programme.

	Workplace-adapted 10-weeks MBSR programme	Original MBSR programme
Duration of programme	10 weeks	8 weeks
Duration of sessions	1.5 h	2.5 h
Total number of sessions	10 sessions	9 sessions
Silent retreat session	Imbedded within the 10 sessions Duration: 1.5 h	Added as the 9th session in the 8-week programme Duration: 7 h

MBSR: mindfulness-based stress reduction.

A workshop on further implementation of mindfulness in the companies was offered to all four companies. The workshop was hosted by hosted by the second author (EGM), the first author (EHB), and an MBSR teacher. At the workshop, participating employee representatives and managers engaged in in-group discussions of *if* they were interested in further implementation of mindfulness and if so, *how* they could imagine this might work best within their company. These in-group discussions led to plenary discussions, and ended with a drafted plan for further implementation of mindfulness in the respective company.

### 2.4. Respondents

Respondents were sampled using the purposive sampling method; Matrix sampling (Campbell et al., 2020). By deploying this method at baseline, EHB reached out to a company representative, typically a person from the management team, and asked this person to invite employees and managers to engage in a focus group interview. The company representative was asked to sample employees and managers that represented both those interested in mindfulness and those not to ensure different perspectives in the focus groups and hence further discussions.

Sampling respondents for the post-intervention focus groups interviews, the Matrix sampling method was again utilized. However, now the MBSR teachers, who had delivered one or more workplace-adapted MBSR programme(s) in the company, were asked to propose employees and managers, who in their opinion would contribute with valuable information regarding the research question. It was made clear to the MBSR teachers that the proposed respondents were to represent both those highly engaged and those who were less engaged during the 10-weeks workplace-adapted MBSR programme. Furthermore, for the post-intervention focus groups, both employees and managers who participated in a 10-week workplace-adapted MBSR programme, non-participants and those who dropped out during a 10-week workplace-adapted MBSR programme were invited to be respondents. In total, 76 respondents participated in a focus group/individual interview at baseline and/or post-intervention. Across companies, 53.9% of respondents were female.

### 2.5. Data collection

Data was collected using semi-structured focus group interviews with 2–5 respondents in each. Focus groups were chosen to enable investigation into the reported individual experiences and shared meaning between respondents. In one of the companies, there was only one manager, and hence, both baseline and post-intervention management interviews in this company was conducted as individual semi-structured interviews. EGM and EHB collected all data, with EGM as the primary moderator and EHB as substitute moderator and observer. In total, 14 baseline interviews (13 focus groups, and 1 individual), and 13 post-intervention interviews (12 focus groups, and 1 individual) were conducted between March 2020 and May 2021. Upon commencement of each interview, respondents were informed about the purpose of the study, their possibility to withdraw at any time, and of the use and storage of data. Oral informed consent was obtained from all respondents.

EGM is an organizational psychologist and researcher. Moreover, EGM is a skilled interviewer and moderator with an extensive amount of experience in establishing safe interview environments. EGM has no previous either personal or professional experience with mindfulness. EHB has an MSc in Public Health, and has both knowledge of, and personal and professional experience with mindfulness and MBIs.

At baseline, the 14 interviews were conducted prior to implementation of the first intervention element. The interviews were performed using a semi-structured interview guide. The interview guide consisted of nine themes, four of which related to workplace social capital and psychological safety: (a) Company prioritization of employee well-being, (b) Collaboration, (c) Tone and communication, (d) The company’s feedback culture. A question related to workplace social capital was, for example, “*How would you characterize working relationships in your company – do you collaborate well or is there sometimes problems?*,” while a question about psychological safety was, for example, “*If something needs to be corrected – or needs to be criticized – how is that done?*”

Post-intervention, the 13 interviews were performed following the workshop on further implementation of mindfulness. However, one of the companies did not wish to participate in such a workshop, and hence, the interviews were conducted following the 10-weeks workplace-adapted MBSR programme. The entire interview guide consisted of eight themes, of which three were related to the workplace social capital and psychological safety: (a) Relations within the company, (b) prioritization of well-being and feedback culture, (c) how employees and managers experience the narrative of the intervention within the company. A question related to workplace social capital was, for example, “*Do you feel that the mindfulness course has affected the way you work together in your company? If yes, how?*,” while a question related to psychological safety was, for example, “*Since the course started, have you then noticed any changes in how you or other people give or receive criticism?*”

Baseline interviews in the four companies took place from February 2020 to November 2020. Post-intervention interviews were conducted from June 2020 to May 2021. Due to the Covid-19 pandemic, two major lockdowns affected this study. Therefore, 19 interviews (70.4%) were performed live online *via* Zoom. These interviews were all recorded using the record function in Zoom and downloaded to a secure drive immediately after the interview. Eight interviews were conducted in-person at the respective workplace sites. These interviews were recorded using Dictaphone and uploaded to the same secure drive and subsequently deleted from the Dictaphone. Throughout the interviews, EHB took notes on atmosphere, sense of tone and appearances, and made initial analytical remarks in the notes.

### 2.6. Analysis

Initially, EHB performed verbatim transcription of all focus group interviews and individual interviews, including noting breaks, length of pauses and tone of voice. Primary analysis was performed using deductive content analysis (Elo and Kyngäs, 2008). This method was chosen because it offers a systematic approach to condensing large amounts of data and enables discussion of possible explanations of why and how mindfulness may impact social capital and psychological safety (Lyhne and Bjerrum, 2021). Hence, two structured categorization matrices were made; one for social capital (Table 2) and one for psychological safety (Table 3) (Elo and Kyngäs, 2008). The matrix for social capital was constructed according to work of Woolcock and Narayan’s on the three types of social capital; bonding, bridging and linking social capital (Woolcock and Narayan, 2000). The matrix for psychological safety was constructed according to the work of Edmondson on psychological safety within teams and organizations (Edmondson, 1999; Nembhard and Edmondson, 2011).

**Table 2:** Categorization matrix, social capital.

	Type of social capital		
	Bonding	Bridging	Linking
What characterizes the experienced social capital of the participating companies?	What characterizes the networks within teams/departments?	What characterizes the networks between teams/departments?	What characterizes the networks between managers and employees?
	What characterizes the norms within teams/departments?	What characterizes the norms between teams/departments?	What characterizes the norms between managers and employees?
	What characterizes the trust within teams/departments?	What characterizes the trust between teams/departments?	What characterizes the trust between managers and employees?

**Table 3:** Categorization matrix, psychological safety.

	Psychological safety		
	To which extend does one feel, he or she can ask for help on a specific problem?	How are mistakes or errors received?	How is the feedback culture; appraisal and criticism?
What characterizes the degree of experienced psychological safety within participating companies?			

Firstly, EHB carefully read through all transcript, and preliminary analytical notes were made. Secondly, meaning units from the transcripts were categorized using the categorization matrices. The categorization matrices were developed using theory of the constructs of workplace social capital (including bonding, bridging, and linking social capital) and psychological safety. The categorization matrices were used to enable identification and extraction of interviewee responses that informed of either the workplace social capital (bonding, bridging, and linking) or psychological safety. Thus, transcripts were read through with the research questions from the categorization matrices in mind. Each time an interviewee response informed of either the workplace social capital or the psychological safety, this meaning unit was extracted. Meaning units were then categorized according to which research question they informed of (workplace social capital, including bonding, bridging, and linking, or psychological safety). For examples of the use of the categorization matrices (see Table 4). EHB and EGM independently categorized a part of the data, and subsequently compared categorized meaning units. Whenever there was divergence in categorization, agreement was reached upon discussion. EHB then conducted the categorization on the rest of the data. Following categorization, inter-coder validation between EGM and EHB was performed. This resulted in an inter-coder agreement of 72.2%. Disagreement was most often caused by differences in interpretation. Hence, agreement was reached on all categorizations upon consultation with notes, full transcripts, and discussion. Throughout the analysis, EGM and EHB remained open to emerging themes of importance to the research question.

**Table 4:** Examples of the use of research questions from the categorization matrices to identify and extract meaning units.

Theoretical construct	Research question	Meaning units
Social capital	What characterizes the networks between teams/departments?	”*[Department X] and [Department Y] had a good collaboration before, (…) but as I say, I also think, it’s become closer, well we talk even more now, eh, and spar a lot more now, I think, during the past three months*” (Male office employee, Company 1)
Psychological safety	To which extend does one feel, he or she can ask for help on a specific problem?	”*Yeah, I think we have become like, a bit more open towards each other, also about things that may be a little vulnerable. That we can use each other. That we can lean on each other”* (Female middle manager, Company 2)

## 3. Results

Firstly, an overview of the baseline social capital and psychological safety in the workplaces will be presented. Secondly, results from the analysis of post-intervention data are presented in three main categories 1. Social capital (with three sub-categories: 1.1. Bonding social capital, 1.2. Bridging social capital, 1.3. Linking social capital), 2. Psychological safety, and 3. Emergent theme: The role of lockdown on the perceived organizational impact of a workplace MBI.

### 3.1. Overview of the social capital and psychological safety at baseline

Pre-intervention focus groups provided insights to the social capital (bonding, bridging and linking) and the psychological safety in the companies at baseline. Across companies, employees and managers indicated a high level of bonding social capital within team/departments, where employees took notice of one another and offered help to those who needed it. A male employee in Company 4 exemplified this:

*“I … just to see if there is anything, we could do to help. Just to ease off … ease off their workload” (Male office worker, Company 4)*

However, the bridging social capital was strained at baseline in all four companies. Across the four companies, employees and managers reported difficulties in the collaboration between departments. Thus, the interdepartmental networks were under pressure. The strained collaborations were mainly centred on a lack of understanding of why the employees in the other departments acted the way, they did, as illustrated by a female employee in Company 3:

*“(…) we’re in the [x department] and those, who are in [y department], (…) we don’t think alike. So, we’re often like … it might be a bit exaggerated, but we don’t understand why they’re not [delivering] what we need” (Female office worker, Company 3)*

Yet, in Company 2, the interviewees expressed that the collaboration between departments was good, illustrated by descriptions of how they would help each other out. Nonetheless, during work intensive times, this ability to help each other appeared to diminish.

The linking social capital was high at baseline in all four companies with managers expressing that they cared about their employees and their well-being. Importantly, employees echoed this experience across companies, especially regarding their immediate manager. Thus, the norm in all four companies was that the management cared about the employees’ well-being, and the employees trusted that their manager did indeed care. In Company 3, however, a female production worker reported not knowing if top management was interested in employee well-being, indicating a lower level of trust between employees and top management:

*“I think, well, I feel, that my immediate manager focusses on it [employee wellbeing, red.], (…) but the top manager, I have no idea, that’s for sure” (Female production worker, Company 3)*

With respect to psychological safety, interviewees from one company expressed that the psychological safety at baseline was high. This high degree of safety was exemplified by feeling safe approaching one’s immediate manager, talking about difficult subjects such as stress as well as acknowledging each other for a job well done. Interviewees from the remaining companies initially reported that it was acceptable to make mistakes and safe to provide negative feedback to colleagues. However, as each interview progressed, interviewees from three companies gave examples of strained psychological safety, for example, that they feared expressing disagreement with the top management, or feared social stigmatization if they violated group norms. Furthermore, two employees at Company 2 independently expressed not wanting to tell anyone at work if they felt stressed, as others might perceive them as being incompetent:

*“I think, if you tell someone that you’re stressed, it’s like saying “I don’t know how to do what I’m doing” (…). I don’t think anyone wants to tell if they’re stressed” (Male employee, Company 2).*

### 3.2. Post-intervention categories

Using deductive qualitative analysis, two main categories and three sub-categories were deduced; (1) *Social capital* with the subcategories: (1.1) *Bonding social capital*, (1.2) *Bridging social capital* and (1.3) *Linking social capital*, and (2) *Psychological safety*. Throughout the analysis, EGM and EHB were open to emerging themes. Thus, an emergent theme resulted in a third main category; (3) *Emergent theme: The role of lockdown on the perceived organizational impact of a workplace MBI.* In only one instance, an employee reported having experienced a potential negative impact on bonding social capital. Neither employees nor managers reported any other potential negative effects. Some respondents reported not having noticed any changes. However, the majority of respondents offered multiple examples of positive changes in both the social capital and psychological safety.

#### 3.2.1. Social capital

##### 3.2.1.1. Bonding social capital

At baseline, employees and managers across companies demonstrated a high degree of bonding social capital, expressed by, for example, helping colleagues within one’s own department. Following the intervention, employees and managers in all four companies reported not having experienced any changes in these regards. However, in Company 1 and Company 4, one or more employees described a positive change concerning their relationship with immediate colleagues, such as, for example, feeling closer to them:

*I (interviewer): “… as a result of this course, have you then become more aware of how your colleagues are doing, or is it the same as before?”*

*IP:“Especially those that I have worked with most. (…) those people, I’m now more in touch with [how they’re doing] (Female office employee, Company 1)*

Results thus indicate that participation in the workplace-adapted MBI may positively influence the bonding social capital at team and departmental level—even when the bonding social capital was high at baseline. This effect was, however, limited to strengthened *networks* within teams or departments. Based on the questions posed, and the interviewees’ responses, there were no indications of changes to norms or trust within teams or departments. Furthermore, as mentioned one employee from Company 4 mentioned frictions between those team members who participated in an MBSR programme, and those who did not:

*”(…) I brought it [further implementation of mindfulness in the organization] up at a Teams meeting, I had with my team, and [I] experienced several people who objected to it and asked how they [non-participants] could be compensated for the time, we [participants] spend practicing mindfulness” (Male office employee, Company 4)*

##### 3.2.1.2. Bridging social capital

Analyses of baseline groups interviews revealed strained interdepartmental collaborations across all four companies, as expressed primarily by an experienced lack of understanding of each other’s work tasks between departments. However, in post intervention interviews, managers and employees across companies expressed that collaboration between departments had increased just as inter-departmental relations had been improved. In Company 1 and Company 4, employees explicitly described how collaboration had improved because of an increase in interdepartmental conversations, which also resulted in constructive discussions concerning work related tasks. A male office employee in Company 1 expressed the following:

*”[Department X] and [Department Y] had a good collaboration before, (…) but as I say, I also think, it’s become closer, well we talk even more now, eh, and spar a lot more now, I think, during the past three months” (Male office employee, Company 1)*

In Company 2 and Company 3, changes directly related to relations between departments were mainly apparent within management groups. Compared to baseline, relations between managers representing different departments improved following the intervention. These changes in bridging social capital indicated a higher level of interdepartmental trust, illustrated by a manager experiencing a greater ability to approach other managers.

*“… well … we can easily walk up to each other and talk. We can come and say:“Hey, do you have five minutes?”, or “I need some help”” (Male middle manager, Company 2)*

Moreover, managers in Company 3 also described an improved interdepartmental collaboration between managers. According to the interviewees, an increased understanding for each other’s work tasks, resulting in better communication and fewer interdepartmental “clashes”, was the main driver of this improvement.

*“I actually think that my work relationship with one of the others, who’s also in the manager group, has improved, where sometimes, we’ve had some misunderstandings or clashes, (…) and he’s gotten a better understanding of the context, I’m a part of” (Female manager, Company 3)*

While the above quote might be analyzed as an expression of bonding social capital within the manager group, it was clear from the interviews that the managers primarily identified themselves as being part of their respective departments, not the management team.

These examples of enhanced bridging social capital—at both employee level and management level—may be felt directly on, for example, improved understanding of each other’s work tasks resulting in improved collaboration, as described above. However, an indirect effect of the intervention on the bridging social capital was evident in all four companies, where participation in the workplace-adapted MBI resulted in an enhanced feeling of knowing one’s colleagues and—as illustrated here—a greater sense of connectedness within the company:

*“I feel, I bring it [mindfulness] with me to work, when I share it with others because of these [mindfulness sessions], and I think, that’s such a good thing to share. Well, we know that we’ve been to the same place. That, I think, actually creates a sense of connectedness” (Female office employee, Company 4)*

Hence, owing to enhanced trust and/or networks between departments, the bridging social capital may be improved between employees as well as between managers through company participation in this organizational level, workplace adapted MBI. Furthermore, this improvement in bridging social capital may be demonstrated both directly through enhanced interdepartmental collaborations as well as indirectly *via* improved interdepartmental connectedness.

##### 3.2.1.3. Linking social capital

At baseline, managers described that the linking social capital was high across all four companies, primarily indicated by a shared norm of caring about one’s employees’ well-being. Employees shared this experience. Hence, room for improvement was small in this regard. Following the intervention, there was still a feeling among employees in all companies, that their managers genuinely cared about the well-being of their employees, and that implementation of the intervention had emphasized this feeling. Moreover, one employee described experiencing his immediate manager as more able to listen to others and generally more caring:

*“now, we have a very strict boss, and she’s very … she’s very strict with us, but as soon as you’ve got something that you want to unload, she’s really sweet and really good at listening. And I don’t know if this mindfulness has made her a better listener, but (…) she seems a lot more loving now and [more] listening” (Male employee, Company 2)*

An enhanced ability to listen to one’s employees may facilitate greater trust between managers and employees. However, the respondents did not directly express this.

Across companies, several managers utilized skills learned through participation in the 10-weeks MBSR programme, such as enhanced awareness, when interacting with their employees, for example in one-to-one conversations:

*“I think, for me, it’s [how mindfulness has affected the way you work] really the way I work with my employees. Well, (…) I continuously try to be aware when I have one-to-ones with them” (Female manager, Company 3)*

This renewed focus on being aware in meetings with one’s employees may foster a strengthened relationship between management and employees. This could happen *via* changes in the norms of how managers and employees engage in these one-to-ones. Thus, participation in this workplace-adapted MBI may impact the linking social capital even in organizations with a high degree of linking social capital at baseline.

#### 3.2.2. Psychological safety

Baseline group interviews revealed that in three of the companies, the psychological safety was strained in some regards. As such, employees reported not wanting to share with managers or colleagues if they felt stressed. Post-intervention interviews pointed to some improvements on psychological safety in these companies. As such, the psychological safety between colleagues appeared to have improved in most of the companies, with interviewees reporting a mutual feeling of being able to share with colleagues how they were feeling or if they had a bad day. Also, they reported being able to bring up difficult topics with colleagues:

*”(…) I feel that I can very easily tell my colleagues if I’m having a bad day (…). I feel like I can share everything with them, actually” (Female employee, Company 2)*

This feeling of psychological safety was also evident among manager colleagues within management, especially in Company 2. Here, the management team had increased their ability to make use of each other’s strengths and actively share experiences:

*“Yeah, I think we’ve become like, a bit more open towards each other, also about things that may be a little vulnerable. That we can use each other. That we can lean on each other” (Female middle manager, Company 2)*

However, a subgroup of employees from one department in one company expressed that that it might not be legitimate to share how they were doing:

*“I don’t think, we do that [share how we’re doing]. This is a … there might be some girls here, but it’s a male dominated workplace” (Female production employee, Company 3)*

The above quote seems to indicate that the interviewed subgroup of women felt that the possible impact of this workplace-MBI on psychological safety may have been hampered as a consequence of the department culture being male dominated. In this particular department, only a small proportion of the employees participated in a 10-weeks MBSR programme. Thus, an additional explanation to the lack of perceived impact on psychological safety might be that only few employees in this department participated in a 10-weeks MBSR programme.

At baseline, interviewees in all four companies described that it was acceptable to make mistakes and to provide feedback to one’s colleagues, which is an indication of high psychological safety. At post-intervention, no changes regarding the acceptability of mistakes or feedback culture were evident in these companies. Yet, as was seen at baseline, employees from one company still expressed a fear of providing feedback to top management:

*”(…) and then I thought that actually I didn’t dare approach her [manager] myself, because I had heard other stories about [how] you got your head ripped off, and that it’s not the easiest conversation to have with her” (Female office employee, Company 1)*

Hence, interviews indicate that company participation in this workplace adapted MBI may impact the psychological safety between colleagues at the same level of employment. However, across the four companies, no impact was evident in the expressed psychological safety between management and employees.

#### 3.2.3. Emergent theme: The role of lockdown on the perceived organizational impact of a workplace MBI

Through the analysis, it became apparent that the lockdowns due to the COVID-19 pandemic may have affected the impact of the intervention on, for example, workplace social capital and psychological safety. The intervention was provided to the four participating companies over the course of 13 months, from March 2020 to April 2021. Hence, the intervention was delivered during several lockdowns due to COVID-19 restrictions. Our analysis gave insights into how these lockdowns may have affected interviewees’ perceptions of the interventions’ impact on social capital and psychological safety. Three out of four companies were particularly affected by lockdowns with employees and managers working from home during the intervention. An objective of this workplace-MBI was to enhance social relations through improved workplace social capital and psychological safety. With employees and managers working from home, and thus being isolated physically from each other, this enhancement in social relations may be challenged, since the amount of social contact was reduced to a minimum. This tendency may also affect how well the impact among participating employees and managers diffuses to the non-participants and thereby the entire organization. A non-participating male employee from Company 3 talked about this potential lack of diffusion:

*“I think that if we’d been together, and we’d sat together in the canteen and the like, well, then there would probably have been some talk [relating to mindfulness]. But seeing we’ve all been isolated, then it becomes very … well … when you’re in a meeting and the like, then it’s only work-related and talk about the things we need to solve” (Male employee, Company 3)*

As such, in these companies, the interventions’ impact on organizational outcomes might in fact be lower than what could have been the case, if employees had been able to meet at work had there not been lockdowns during the intervention period. However, the effect of COVID-19-lockdowns on this study’s results remains unknown and a cause for speculation.

## 4. Discussion

The main purpose of this study was to examine the potential impact of an organizational-level workplace-MBI including a workplace-adapted MBSR programme on social capital and psychological safety. By applying deductive content analysis to the transcribed pre and post intervention focus group interviews, we gained insight into how this intervention could potentially impact the psychosocial work environment following changes in the social capital and psychological safety.

In this study, interviewees expressed a high degree of both bonding and linking social capital at baseline, leaving only a small room for improvement within these domains. However, the bridging social capital was strained in all four companies. Post-intervention data indicate that the bridging social capital may have been improved across companies, and that both managers and employees reported experiencing small positive changes to the bonding and linking social capital. The psychological safety was somewhat strained at baseline in three of the included companies. Post intervention, the psychological safety at the same level of employment—manager to manager or employee to employee—appeared enhanced.

Albeit the research area of mindfulness in the workplace is a budding field, the impact of mindfulness on specific psychosocial factors, such as social capital and psychological safety, is an even more unchartered territory. However, the results of the present study indicate changes in these two theoretical concepts following a workplace-MBI including a workplace-adapted MBSR programme. Thus, company participation in this intervention may have the potential to enhance workplace relations affecting the social capital and psychological safety in the workplace. Previous research on mindfulness in workplace settings has found similar positive relational effects. Hence, mindfulness training has been found to foster intergroup prosocial behaviour (Berry, 2017; Sajjad and Shahbaz, 2020). The promotion of such behavior is proposed to be facilitated *via* enhanced empathy and reduced tendency to engage in “them” versus “us”-thinking (Berry, 2017). Similar to the present study, such prosocial behaviour may result in enhanced interdepartmental collaboration, understanding and trust. Moreover, research on mindfulness in workplace settings has demonstrated associations between high levels of mindfulness and lower levels of enacted incivility at work (Hülsheger et al., 2021) as well as less moral disengagement (Brendel and Hankerson, 2021). Minimizing these negative relational characteristics may in effect enhance the psychosocial work environment and hence improve the mental well-being of employees and managers. In the present study, managers expressed being more aware and listening when they engage in conversation with their employees. In a qualitative study of a workplace-MBI on leader capabilities by Rupprecht and colleagues, the authors found similar results (Rupprecht et al., 2019). Similar to the present study, Rupprecht et al. (2019) found that managers experienced enhanced abilities to listen actively when engaging in conversations, and greater ability to maintain their attention during social interactions, such as in meetings. Hence, the relational impact of this workplace-MBI including a workplace-adapted MBSR programme is in line with previous research. Moreover, the quality of interpersonal relations—also in the workplace—impacts greatly on mental health and well-being (Holt-Lunstad et al., 2010; Teo et al., 2013; Santini et al., 2015; Dahl et al., 2020; Roffey, 2021; WHO, 2022). Therefore, the relational effects regarding especially bridging social capital and psychological safety between same-level colleagues may have the potential to contribute to improved mental health of employees and managers in workplaces.

It is noteworthy that MBSR is a complex intervention consisting of a number of activities. Hence, MBSR includes, for example, both the active ingredient, practice of mindfulness, and a group-based approach. One might argue that positive changes in the workplace social capital and psychological safety might have been brought about by simply creating a space for employees and managers to interact outside regular work related meetings or the likes. Put differently; might the same results have been obtained without the active ingredient, that is mindfulness? With no active control group, this question will inevitably remain unanswered. Nevertheless, according to mindfulness theory and previous research, mindfulness is linked to enhanced relational outcomes by means of, for example, increased self-regulation, attention, active listening as well as understanding and compassion for others (Glomb et al., 2011; Kabat-Zinn, 2013; Good et al., 2015; Rupprecht et al., 2019; Dahl et al., 2020). Some of these underlying competencies are also evident in the results of the present study, for example, improved active listening and understanding for and of others. Also demonstrated in another study from the present research project, participation in this workplace-MBI may improve the mental health skills of employees and managers (Bonde et al., 2022). Mental health skills are here understood as skills that serve as protection of one’s mental health, such as emotion regulation, and engagement in social relations (WHO, 2012). Findings from that study indicate that following this workplace-MBI, employees and managers may develop an increased awareness of how others perceive things differently from one self and be more responsive instead of reactive in social interactions (Bonde et al., 2022). These acquired skills are thus also in line with mindfulness theory and previous research. Therefore, it seems unlikely that the impact on workplace social capital and psychological safety could have been obtained without the active mindfulness component. However, the group-based mode of delivery is an intrinsic part of the MBSR programme, and hence, results from following an MBSR programme entails effects related to the intervention being group based (Mccown et al., 2010; Kabat-Zinn, 2013). Adding to this, the explicit focus on creating safe and trusting group environments that facilitates sharing of experiences, may also serve as an important component of the intervention to impact social relations in the workplace. This only strengthens the notion that MBSR may be merited even more in organizations, such as workplaces, where relations are of long duration and of great importance to our well-being.

Through the analysis it became apparent that the Covid-19 pandemic and the lockdowns resulting thereof might have affected the diffusion of organizational effects, causing potential dilution of the impact on workplace social capital and psychological safety. However, a more critical theoretical stance could be that the effects might be magnified by the lockdowns. This could be the case if colleagues had been separated for longer periods of time, and that simply re-connecting with one’s colleagues might cause the perceived impacts on the workplace social capital and psychological safety. Yet, referring to the above argument that specific competencies related to mindfulness theory and findings from previous research (Glomb et al., 2011; Kabat-Zinn, 2013; Good et al., 2015; Rupprecht et al., 2019; Dahl et al., 2020) are evident in the results of this present study, this is deemed unlikely.

### 4.1. Strengths and limitations

This study was conducted in a close collaboration between mindfulness experts and an experienced work and organizational psychologist. Therefore, in-depth knowledge of both mindfulness and workplaces where represented in the research group, ensuring that both knowledge of the intervention, mechanisms and, context were sufficiently represented. Furthermore, one of the main strengths of this study was that the intervention was offered to all employees and managers in the respective four companies. By deploying this population-based approach, no groups were singled out as having a special need for this intervention, and were thus not stigmatized (Rose et al., 2008). Also, the workplace-adapted MBSR programme was systematically developed using best practice when adapting MBIs to specific contexts (Crane et al., 2017). Moreover, the study includes data from 76 respondents from four companies representing different business areas with interviews from both baseline and post-intervention. This has resulted in a large data material allowing for thorough understanding of how the social capital and psychological safety may be impacted by this workplace-MBI across business areas. Lastly, EGM did not have any pre-existing experience with mindfulness, neither personal nor professional. Hence, close collaboration between EGM and EHB ensured that the analysis did not rely on a preunderstanding of how mindfulness might impact psychosocial factors such as social capital and psychological safety. Four companies representing different business areas were included in this research project. Similar patterns in impact on workplace social capital and psychological safety were seen across companies. Hence, this may indicate that the results presented in this study are not limited to specific companies or business areas.

Still, the included companies were all self-selected, and chose to either actively seek out to be part of the research project or expressed interest upon direct contact from a representative of the research group. Thus, the results of this study may be restricted to companies with a preceding interest in mindfulness or mental health promotion. Moreover, interview questions relating to workplace social capital and psychological safety could have benefitted from being more systematically included in the interview guide. As such, questions related to workplace social capital might, for example, have been structured in interview questions divided into networks, norms, and trust. Yet, the interview guide was formulated to capture psychosocial factors such as workplace social capital and psychological safety in broad terms and therefore captured essential data needed for interpreting the impacts on these two theoretical concepts. Furthermore, by the words of Edmondson & Lei “… *psychological safety is essentially a group-level phenomenon*” (Edmondson and Lei, 2014). Hence, the psychological safety may vary across teams, departments, between managers and employees and so on. Thus, it may be problematic to conclude on the psychological safety for an entire organization, since this entails multiple teams and levels of hierarchy. Therefore, the analysis of psychological safety would have benefitted from data collected within teams with several team members from each team. Instead, focus groups in this study consisted of, respectively, employees and managers from different teams and departments. Thus, this study does not provide information of the impact of this workplace-adapted MBI on team psychological safety. Hence, future research may benefit from including focus group interviews within teams. However, it is unknown whether employees and managers in this study intuitively provided answers based on their experiences within their respective teams when engaging in a focus group interview. Moreover, the majority of focus group interviews were conducted live online *via* Zoom. This digital format made it difficult to interpret body language and inter-respondent interactions when respondents were not in the same room. However, beyond the difficulties in interpreting these non-verbal interactions, EGM and EHB did not experience any complications conducting focus groups live online. Yet, conducting focus group interviews live online does have potential positive aspects (Flayelle et al., 2022). By using the online format, we were able to reach more respondents and gain access to international managers and employees, we normally would not have had access to (Flayelle et al., 2022). Furthermore, the online format was both time and cost effective and allowed for EGM and EHB to communicate occasionally *via* the chat function when needed during the interviews (Flayelle et al., 2022). Lastly, in this study, one employee reported having experienced some negative effects in the bonding social capital relating to frictions between participants and non-participants within his department. Such frictions could cause disruption in the networks within a team or department and thus possibly negatively affect the bonding social capital if not dealt with properly. These frictions may pose a barrier to further implementation of mindfulness in an organization. Therefore, future research ought to investigate facilitating and obstructing factors that may influence the impact of a workplace-adapted MBI.

### 4.2. Implications and perspectives

According to the results of the present study, the utilized MBI seems to have a potential for facilitating a positive impact on workplace social capital as well as psychological safety among people at the same level of employment. Thus, this study contributes with knowledge to the budding field of potential organizational impacts of MBIs delivered in a workplace setting. Hence, this study adds to the notion that mindfulness training in a workplace setting not only has the capacity to improve individual well-being or mental health skills (Good et al., 2015; Vonderlin et al., 2020; Bonde et al., 2022), but that it may also have the potential to contribute to improved psychosocial work environments. For employees and managers workplace-MBIs may lead to improved mental health skills (Bonde et al., 2022). For organizations, workplace-MBIs may contribute to healthier psychosocial work environments adding to improved individual mental well-being (WHO, 2012; Mikkelsen et al., 2020; WHO, 2022). Furthermore, the study provides additional knowledge of the ways that MBIs may affect the psychosocial work environment. These insights may be used for developing program theories for future research both in the fields of mindfulness, and work and organizational psychology. Future research would benefit from investigating barriers and facilitators to implementing mindfulness in workplace settings in order to gain insight into what works for whom and under what circumstances.

## 5. Conclusion

The aim of this study was to investigate how an organizational-level workplace-MBI including a workplace-adapted MBSR programme may impact on workplace social capital and psychological safety, potentially leading to improved individual mental health. Compared to baseline, a positive impact on especially the bridging social capital was seen in all included companies. Moreover, small positive changes to the psychological safety between people at the same level of employment were uncovered. The perceived impact may be affected by the COVID-19 pandemic and following lockdowns. However, it is deemed unlikely that this would lead to an exaggeration of the intervention impact. Thus, this workplace-MBI appear to have a positive impact on workplace social capital and psychological safety, which may in turn contribute to improved mental well-being of employees and managers. However, even though the study included companies representing different business areas, the results may be limited to companies that have a pre-existing interest in either mindfulness or workplace well-being. Future research should include a range of different types of companies, and investigate facilitators and barriers of implementing mindfulness-based interventions in workplace settings.

## Data availability statement

The raw data supporting the conclusions of this article will be made available by the authors, without undue reservation.

## Ethics statement

Ethical review and approval was not required for the study on human participants in accordance with the local legislation and institutional requirements. Written informed consent for participation was not required for this study in accordance with the national legislation and the institutional requirements.

## Author contributions

LJ and EGM designed the study. The interview guide was developed by EGM and EHB, who also collected the data analyzed in the present study. EHB was responsible for the transcription of the interviews. EGM and EHB performed initial coding of part of the data. EHB subsequently performed coding and categorization of all the data. In collaboration, EGM and EHB performed inter-coder reliability tests to ensure validity of the categorization. EGM and EHB were in continuous dialogue through the analysis process. The final results were discussed with LJ and LOF. LJ, LOF, EGM, and EHB collectively decided on the focus of the present publication. EHB drafted the manuscript, while EGM, LJ, and LOF made invaluable comments and corrections. All authors contributed to the article and approved the submitted version.

## Funding

This study was funded by The Velliv Association. Award number: 19-0506.

## Conflict of interest

The Danish Center for Mindfulness, Department of Clinical Medicine, Aarhus University offers revenue-funded MBSR programmes and MBSR teacher training.

The authors declare that the research was conducted in the absence of any commercial or financial relationships that could be construed as a potential conflict of interest.

## Publisher’s note

All claims expressed in this article are solely those of the authors and do not necessarily represent those of their affiliated organizations, or those of the publisher, the editors and the reviewers. Any product that may be evaluated in this article, or claim that may be made by its manufacturer, is not guaranteed or endorsed by the publisher.
